# Scaffold proteins of cancer signaling networks: The paradigm of FK506 binding protein 51 (FKBP51) supporting tumor intrinsic properties and immune escape

**DOI:** 10.32604/or.2023.028392

**Published:** 2023-06-27

**Authors:** LAURA MARRONE, MASSIMO D’AGOSTINO, CAROLINA GIORDANO, VALERIA DI GIACOMO, SIMONA URZINI, CHIARA MALASOMMA, MARIA PAOLA GAMMELLA, MARTINA TUFANO, SIMONA ROMANO, MARIA FIAMMETTA ROMANO

**Affiliations:** 1 Department of Molecular Medicine and Medical Biotechnology, University of Naples Federico II, Naples, 80131, Italy; 2 Dipartimento di Diagnostica per Immagini e Neuroradiologia, Fondazione Policlinico Universitario “A.Gemelli” IRCCS, Università Cattolica S. Cuore, Rome, Italy

**Keywords:** Immunophilin, Alternative splicing, Signal transduction

## Abstract

Scaffold proteins are crucial regulators of signaling networks, and their abnormal expression may favor the development of tumors. Among the scaffold proteins, immunophilin covers a unique role as ‘protein-philin’ (Greek ‘philin’ = friend) that interacts with proteins to guide their proper assembly. The growing list of human syndromes associated with the immunophilin defect underscores the biological relevance of these proteins that are largely opportunistically exploited by cancer cells to support and enable the tumor’s intrinsic properties. Among the members of the immunophilin family, the *FKBP5* gene was the only one identified to have a splicing variant. Cancer cells impose unique demands on the splicing machinery, thus acquiring a particular susceptibility to splicing inhibitors. This review article aims to overview the current knowledge of the *FKBP5* gene functions in human cancer, illustrating how cancer cells exploit the scaffolding function of canonical FKBP51 to foster signaling networks that support their intrinsic tumor properties and the spliced FKBP51s to gain the capacity to evade the immune system.

## Introduction

Scaffold proteins are crucial regulators of signaling networks [1,2]. In addition to interacting with multiple members of a signaling pathway and tethering them into complexes [2], these multidomain proteins can exert allosteric control over their partners and are themselves the target of the regulation [2]. With their protein-protein interaction modules, scaffold proteins assist the assembly of intracellular signaling complexes downstream of numerous receptors [3]. Abnormal expression of scaffold proteins may contribute to the dysregulation of signaling pathways and favor the development of tumors [4,5].

Relevant roles in cancer have been reported for the Cas family member proteins [6], particularly, NEDD9 [7] assembles complexes involving oncogenic kinases, including focal adhesion kinase (FAK), Abelson tyrosine-protein kinase (ABL), Rous sarcoma tyrosine-protein kinase (SRC), and Aurora-A (AURKA) thus [7], regulating the magnitude and duration of cell signaling cascades acting in tumorigenesis and metastases. The scaffold protein Ezirin, a member of the ezrin/radixin/moesin (ERM) family of proteins [8], acts as a cross-linker of membrane proteins or phospholipids in the plasma membrane and the actin cytoskeleton and functions as a platform for signaling molecules at the cell surface [8]. As focal points for the association of signaling molecules and downstream pathways, scaffold proteins are explored as potential anticancer therapeutic targets [8–13]. RAC (Rho family)-alpha serine*/*threonine*-*protein kinase (AKT), the well-known transducer of oncogenic signals in virtually all human tumors [14], has received considerable attention from a therapeutic perspective [15].

The scaffold protein sodium-hydrogen exchanger regulatory factor 1 (NHERF1) is being explored to optimize current anticancer drugs targeting EGFR signaling [16] because it controls EGFR recycling/degradation. Stabilizing the EGFR on the plasma membrane, NHERF1 increases cell sensitivity to the tyrosine kinase inhibitors that affect EGFR-driven motility and invadopodia-dependent ECM proteolysis in cancer cells [16].

Within the scaffold proteins, immunophilins cover a unique role as “protein-philins” (Greek “philin” = friend) that interact with proteins to guide their proper assembly [17–19]. Immunophilins include two large subfamilies of proteins, namely cyclophilins and FK506 binding proteins (FKBPs) [18] that were first identified as the binding proteins for the immunosuppressant cyclosporin (a cyclophilin ligand) and FK506 and rapamycin (FKBP ligand) [18]. The growing list of human syndromes associated with the immunophilin defect underscores the biological relevance of these proteins [20–23]. Immunophilins are largely opportunistically exploited by cancer cells to support and enable the tumor’s intrinsic properties [23–30]. For a comprehensive review of immunophilins as dynamic scaffolding elements in diverse signaling complexes, see [19].

Among the members of the immunophilin family, the *FKBP5* gene was the only one identified to have a splicing variant [31]. Isoform 1 of the *FKBP5* gene contains a tandem FK separated by a short linker sequence and three tetratricopeptide repeat motifs (TPR) for protein/protein interaction. The N-terminal FK is responsible for the peptidylprolyl-isomerase (PPIase)- and ligand-binding activities. The 2^nd^ FK is inactive as PPIase but retains an interaction ability. This domain contains an ATP/GTP-binding sequence. For the structure of FKBP51, we refer to the original papers by Sinars et al. [32], and Bracher et al. [33].

The spliced isoform 2 of the *FKBP5* gene, FKBP51s, was first identified in melanoma patients; it is codified by the transcript variant 4 (NM_001145777.1 mRNA). Because of a frameshift, FKBP51s has multiple differences in the coding region and 3′ UTR compared to canonical variant 1. The resulting protein (NP_001139249.1) is shorter because it lacks the TPR protein/protein interaction domain, and has a distinct C-terminus compared to canonical isoform 1 [31].

Alternative splicing of mRNA precursors is an almost ubiquitous and extremely flexible gene checkpoint in humans [34]. It allows cells to create protein isoforms of different, sometimes opposite functions from a single gene. Cancer cells actively exploit alternative splicing to expand their proteome to accomplish the diversity of their hallmarks, including survival, proliferation, migration invasion, renewal, and immune evasion. Many isoforms produced in this way are developmentally regulated and preferentially re-expressed in tumors [34].

Due to its multiple interactors for which we refer to Hähle et al. [35], FKBP51 has pleiotropic functions and regulates numerous fundamental aspects of cell biology and physiology of living organisms, including development [36], differentiation [37,38] metabolism [39], response to hormones [32] and immune response [40]. Our review focuses on the scaffold roles of FKBP51 involved in signal transduction pathways and genetic and epigenetic regulation that drive cancer initiation and progression. We illustrate how cancer cells exploit the canonical FKBP51 to foster signaling networks, as, for example, NF-κB, Akt, and TGF-β that support tumor intrinsic properties and the spliced FKBP51s to gain the capacity to evade the immune system.

## FKBP51 Scaffold Roles in Signal Transduction

### Nuclear factor-kappaB (NF-κB) signaling

The NF-κB signal transduction pathway is the prototype of modular composites of functionally interdependent sets of proteins that coordinate to translate environmental stimuli into a cellular response [41]. In 2004, Bouwmeester et al. characterized the protein interaction network of Tumor necrosis factor (TNF)-α/NF-κB pathway components using an integrated approach comprising tandem affinity purification, liquid-chromatography tandem mass spectrometry, network analysis, and directed functional perturbation studies using RNA interference [41]. Such a mapping study identified FKBP51 for the first time as a major multifunctional kinase cofactor in NF-κB signaling among 221 molecular associations and 80 unknown interactors. This latter was co-purified with IκB kinase (IKK)α, IKKε, transforming growth factor-β-activated kinase 1 (TAK1), and mitogen-activated protein kinase kinase-1 (MEKK1). The interaction with IKKα was confirmed by co-immunoprecipitation. Following RNA interference of FKBP51, the authors demonstrated an impaired NF-κB activation, measured in a luciferase reporter assay, underlining an essential role for the immunophilin in the overall signaling process of NF-κB activation.

In 2014, Erlejman et al. [42] reported that FKBP51 impaired the nuclear translocation rate of NF-κB and its transcriptional activity. Because FKBP51 and FKBP52 are responsible in a mutually exclusive fashion for the retro-transport mechanism of steroid hormone receptors, the authors hypothesized that these immunophilins could similarly regulate the translocation of NF-κB proteins. They found an association of either FKBP51 or FKBP52 with NF-κB/RelA, with FKBP52 promoting the nuclear translocation while FKBP51 the cytoplasmic retention. Moreover, following Chromatin immunoprecipitation (ChIp) assays, they proposed that both immunophilins are recruited to the promoter sequences of NF-κB regulated genes, with FKBP51 inhibiting NF-κB transcriptional activity, while FKBP52 favors such activity.

**Figure 1:**
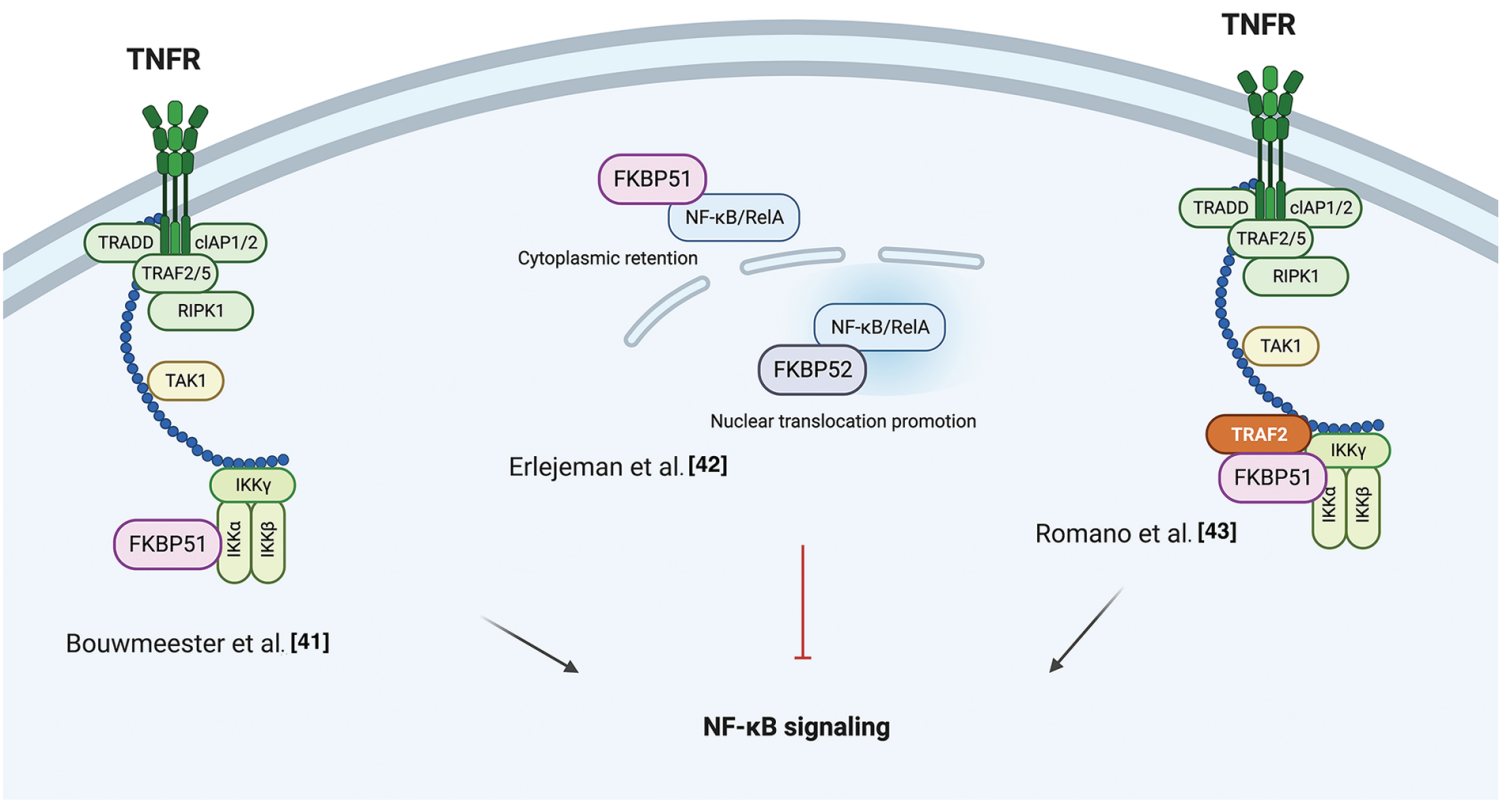
*FKBP51 in the NF-κB signaling*. Left, according to Bouwmeester et al. [41], FKBP51 is the major interactor of IKKα essential for activation of the transcription factor. Middle, as to Erlejeman et al. [42], FKBP51 impairs the nuclear translocation rate of NF-κB and its transcriptional activity. Right, Romano et al. [43] show FKBP51 guarantees the assembly and activity of the IKK complex. By interacting with TRAF2, it reinforces IKK recruitment to K63 ubiquitin chain and keeps closer RIP1, TAK1, and IKK. The illustration was started from scratch, created with BioRender.com original design.

In 2015, Romano et al. [43], by co-immunoprecipitation assays, confirmed the physical interaction between FKBP51 and IKKα and also demonstrated an interaction with IKKγ, IKKβ, and TNF-receptor associated factor 2 (TRAF2). FKBP51-knockdown inhibited the binding of IKKγ to the IKK catalytic subunits, i.e., IKKα and IKKβ, and impaired overall IKK catalytic activity. Either FKBP51 TPR and PPIase domains were required for their interaction with TRAF2 and IKKγ, whereas only the TPR domain was involved in interactions with IKKα and β. TRAF2 catalyzes K63-linked polyubiquitination of receptor-interacting protein 1 (RIP1) in response to TNFα receptor triggering; this non-canonical polyubiquitin chain recruits both TAK1 complex (consisting of TAK1 and TAK1-binding proteins TAB2, and TAB3) and the IKK complex, by binding directly to the ubiquitin-binding domains present on TAB2 and IKKγ. By interacting with TRAF2, FKBP51 reinforces IKKγ recruitment to the K63 ubiquitin chain that keeps closer RIP1, TAK1, and IKK [44]. FK506 and SAFits, respectively, unselective and specific inhibitors of the FKBP51 isomerase activity, impaired the IKK-regulatory role of FKBP51. These results support the conclusion that FKBP51 promotes NF-κB activation by serving as an IKK scaffold and an isomerase. Fig. 1 summarizes the different results from literature on FKBP51 in the NF-κB signaling.

### AKT signaling

A role for FKBP51 as a scaffold protein in AKT signaling was proposed in 2009 by Pei et al. when they identified the immunophilin as part of the protein complex AKT/PH domain leucine-rich repeat protein phosphatase (PHLPP), which enhanced the de-phosphorylation of AKT in a pancreatic cancer context [45]. The authors co-immunoprecipitated PHLPP1 and AKT with FKBP51, showing that the immunophilin, AKT, and PHLPP exist as a complex in cells. By employing FKBP51 mutants, they demonstrated that the N-terminus of the immunophilin (FK1 and FK2 domains) is bound to Akt, while the C-terminus, i.e., TPR domain, to PHLPP. They observed that, in the absence of FKBP51, Akt was hyperphosphorylated at Serine 473 (S473) due to the inefficient binding of PHLPP to Akt. Moreover, they found that AKT S473 phosphorylation was conserved upon the overexpression of the FKBP51 mutant FD67/68DV that lacks peptidylprolyl isomerase activity, suggesting that FKBP51 regulates AKT phosphorylation in an isomerase-independent manner and, thus, acting as a scaffolding protein to promote the AKT-PHLPP interaction [45]. Fabian et al. performed a detailed analysis of the domains involved in the interaction between FKBP51 and AKT, showing that AKT could directly bind to FKBP51 via the FK1 domain or indirectly via the TPR domain through heat-shock protein 90 (HSP90) [46]. Notably, other parts of the FK1 domain that do not exert PPIase activity are involved in the binding of FKBP51 to AKT [46]. Mutations in the FK1 site abolished the isomerase activity without affecting binding to AKT [46]. In 2017, the research group that first identified the role of FKBP51 as a scaffold protein for PHLPP [45] reported that this scaffold does not necessarily assist the de-phosphorylation of AKT [47]. They identified the sirtuin 7 (SIRT7) as an interactor of FKBP51 and responsible for its deacetylation at residues K28 and K155. They found that in different breast or prostate cancer cell lines, depletion of SIRT7 significantly increased the phosphorylation of AKT at S473 by regulating the acetylation status of the immunophilin [47]. Moreover, conversely, the FKBP51 acetylation was increased by P300/CBP and produced an increased AKT phosphorylation at S473 [47].

Gassen et al. reported a role for FKBP51 in autophagy regulation that involved AKT1 and PHLPP. They found a physical interaction between FKBP51 and Beclin-1 that increased the stability of Beclin-1 [48]. They showed that FKBP51 recruits E3 ubiquitin ligase S-phase kinase-associated protein 2 (SKP2) to Beclin-1. SKP2 executes K48-linked ubiquitination at K402 of Beclin-1, resulting in proteasomal degradation. The authors propose that the recruitment of SKP2 to FKBP51 produced defective K48-linked ubiquitination of Beclin-1 due to PHLPP-mediated AKT deactivation, which hampered protein degradation.

**Figure 2:**
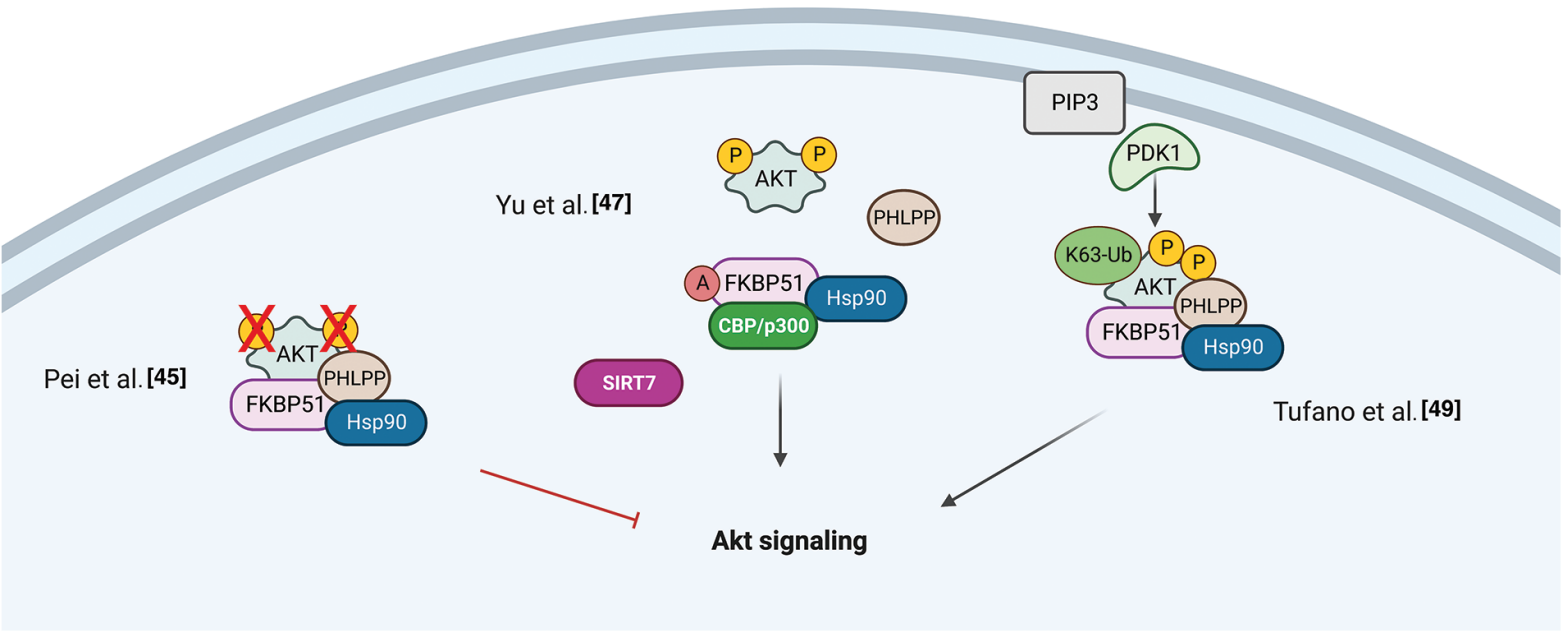
*FKBP51 in the AKT signaling*. Left, FKBP51 is a scaffold protein for the complex AKT/PHLPP and promotes AKT inactivation in a pancreatic cancer context [45]. Middle, FKBP51 acetylation status influences its binding to the AKT/PHLPP complex. When bound to SIRT7 is deacetylated and binds AKT and PHLPP. When FKBP51 is acetylated by P300/CBP, AKT phosphorylation increases as the complex is disrupted [47]. Right, the FKBP51/Akt/PHLPP complex operates in K63-ubiquitination of Akt, thus supporting the phosphorylation of Akt [49]. The illustration was started from scratch, created with BioRender.com original design.

A very recent paper by our group demonstrated that the TPR domain of FKBP51 mediates AKT ubiquitination at K63, which is an essential step for AKT activation [49]. The spliced FKBP51, lacking such a domain, could not link K63-Ub residues to AKT. PHLPP stabilized the level of E3-ubiquitin ligase TRAF6 and supported K63-ubiquitination of AKT. This finding introduces an unknown oncogenic role for the phosphatase PHLPP supported by the interactome profile of FKBP51 carried out on melanoma cells overexpressing PHLPP [49]. An over-representation of components involved in cell division, transcription, and translation, along with a decrease of those related to apoptosis, highlighted a relevant role for PHLPP in improving oncogenic hallmarks. The spliced FKBP51 isoform can explain the discrepant results from the literature. Accelerated splicing towards the FKBP51s could shift the equilibrium between the two isoforms generating a reduced ability to ubiquitinate AKT due to the loss of the TPR domain. Also, a low tumor level of PHLPP, as it may occur in pancreatic cancer [49], can lead to a scarce activation of AKT on FKBP51-overexpression. Fig. 2 summarizes the different results from literature on FKBP51 in the AKT signaling.

### AMPK/mTOR signaling

**Figure 3:**
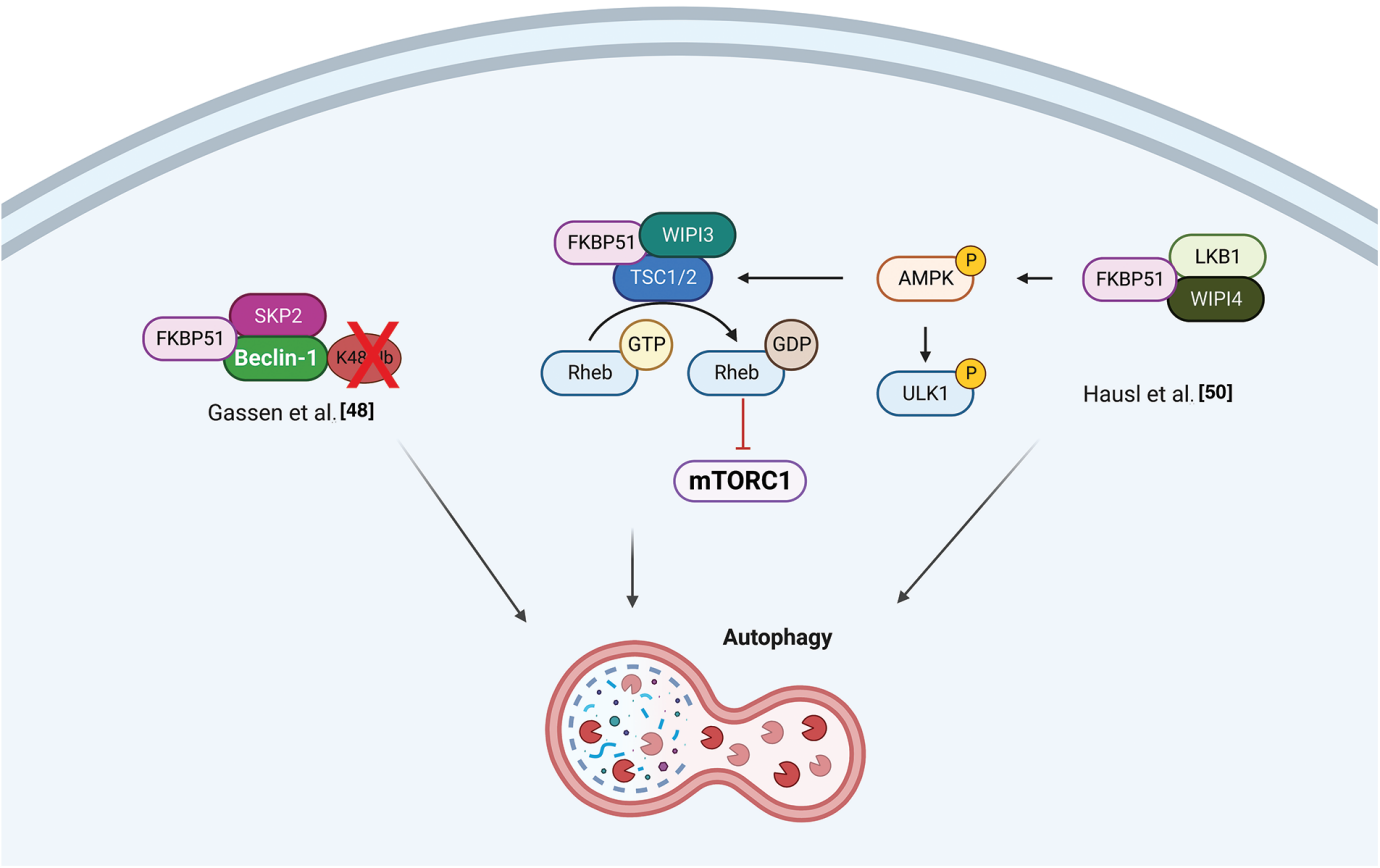
*FKBP51 in the AMPK/mTOR/autophagy axis*. Left, FKBP51 recruits LKB1 to the WIPI4-AMPK regulatory platform to induce AMPK phosphorylation and autophagy initiation by direct phosphorylation of the ULK1 complex. Furthermore, FKBP51 interacts with TSC2, in a WIPI3-dependent manner, and activates autophagy through mTOR inhibition [50]. Right, FKBP51 can directly act on autophagy by binding Beclin-1 and producing a defective K48-linked ubiquitination of Beclin-1 by SKP2 [48]. The illustration was started from scratch, created with BioRender.com original design.

Recent findings position FKBP51 as a central regulatory switch between AMPK and mTOR, pivotal proteins in autophagy regulation [48,50]. The balance between the adenosine 5′-monophosphate (AMP)–activated protein kinase (AMPK) and the mechanistic target of rapamycin (mTOR) regulates autophagy. WD-repeat protein interacting with phosphoinositides (WIPI) proteins are essential scaffold proteins that link autophagy’s key regulatory elements with metabolite-sensing enzymes [51]. Following Bakula et al., who demonstrated that WIPI3 and WIPI4 are essential scaffolders of the liver kinase B1 (LKB1)/AMPK/Tuberous Sclerosis (TSC)1/2 signaling network [51], Häusl et al. [50] found that FKBP51 recruits LKB1 to the WIPI4-AMPK regulatory platform to induce AMPK phosphorylation, which stimulates autophagy initiation by direct phosphorylation of UNC51-like kinase 1 (ULK1) complex, one of the most upstream components acting in the autophagy machinery. Furthermore, performing co-immunoprecipitation studies in neuronal cells, the authors showed an association of FKBP51 with TSC2, which depended on the presence of WIPI3, opening the possibility of an inhibitory role of FKBP51 in mTOR signaling. Fig. 3 illustrates the proposed mechanisms for FKBP51 roles in the AMPK/mTOR/autophagy axis.

### Transforming growth factor-β1 (TGF-β1)/EMT signaling

Using a differential display technique, Giraudier et al. found increased expression of FKBP51 in megakaryocytes of patients suffering from idiopathic myelofibrosis [52]. The same group demonstrated that FKBP51 sustained the production and release of TGF-β1 in the bone marrow microenvironment of a murine model of myelofibrosis mediated by NF-κB activation [53]. These results highlighted for the first time a pathogenetic role for FKBP51 in such a myeloproliferative disorder characterized by fibrosis development [53]. The FKBP51 stimulating effect on TGF-β1 production was confirmed by Romano et al. in melanoma [54]. They also demonstrated a role for FKBP51 in regulating the TGF-β signaling [55]. By co-immunoprecipitation assays, Romano et al. found that FKBP51 interacts with the general transcriptional co-activator p300 and the TGF-β transcription factors Smad2/3 [55] and promotes some transcriptional activities of the TGF-β, precisely the gene expression of vimentin (VIM) and secreted protein acidic and cysteine-rich (SPARC), associated with accelerated tumor growth, invasion, and poor prognosis of melanoma [55]. ChIp assays showed FKBP51/p300 complexes bound to the promoter of the melanoma cancer stem-cell marker ATP-binding cassette transporter G2 (ABCG2) [54]. This finding, together with the observation of an increased transcript level of ABCG2 and an increased number of ABCG2+ cells upon FKBP51 overexpression in melanoma cells, further supported a role for FKBP51 as a co-regulator in gene expression [54]. Interestingly, ABCG2+ melanoma stem cells showed the highest expression levels of epithelial to mesenchymal transition (EMT) genes, including Twist basic helix-loop-helix transcription factor 1 (TWIST), Snail family of zinc-finger transcription factors (SNAIL), Snail Family Zinc Finger 2 (SLUG), CDH-2/N-cadherin, VIM, SPARC [54] highlighting EMT and cancer stemness are intertwined aspects in melanoma biology and progression.

**Figure 4:**
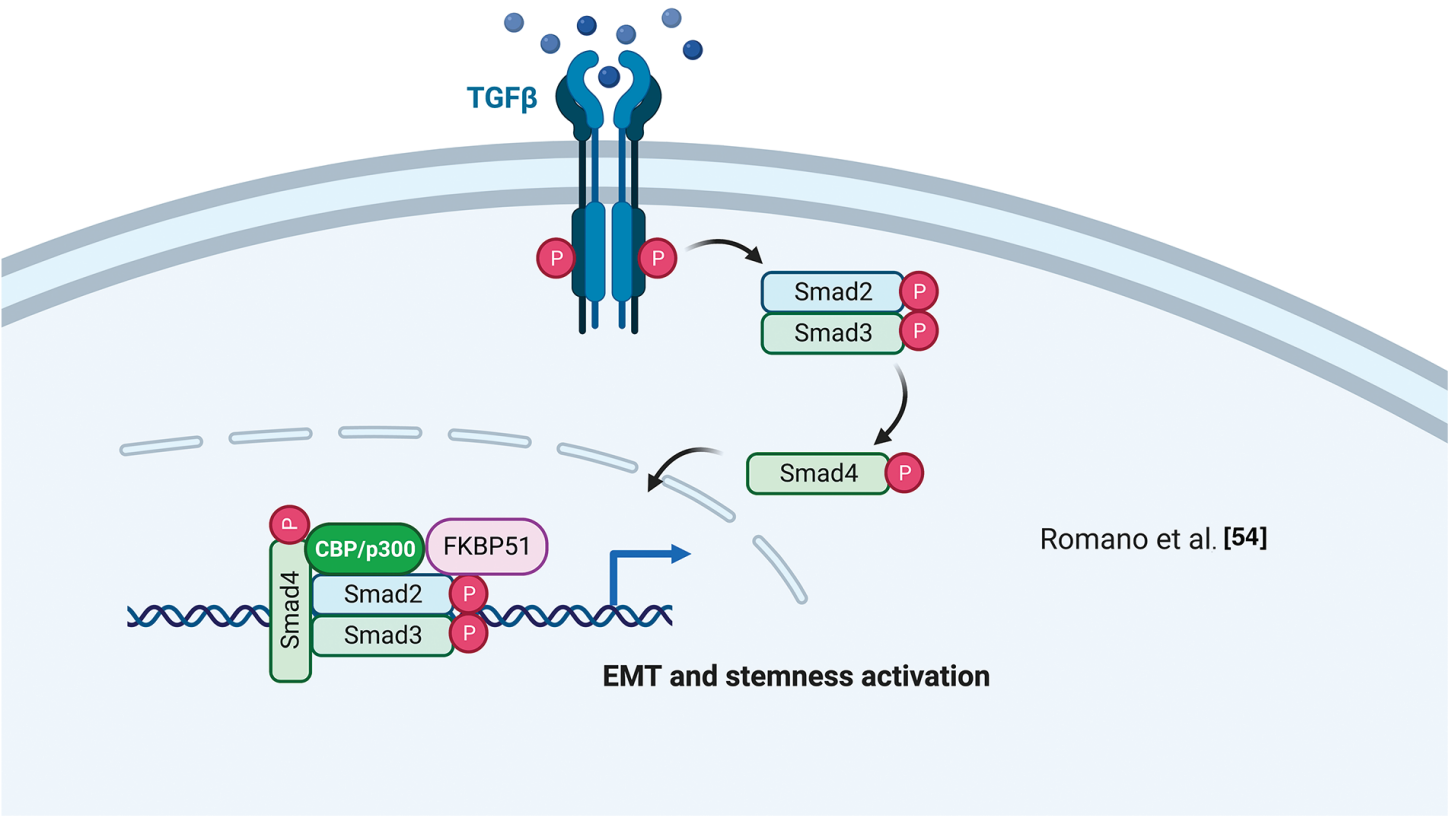
*FKBP51 in the TGF-β signaling*. FKBP51 forms a complex with P300 and Smad2/3 that binds to the promoters of genes involved in cancer stemness and EMT. The illustration was started from scratch, created with BioRender.com original design.

By a cell-by-cell immunohistochemical analysis in colorectal cancer (CRC) and liver metastases resected after chemotherapy with oxaliplatin, Rotoli et al. showed a molecular interaction between the scaffold proteins angiomotin-like 2 (AmotL2), FKBP51, and IQ motif containing GTPase-activating protein 1 (IQGAP1) proteins within the EMT context of cancer tissues [56]. The colocalization of CD34 (a telocyte marker) with AmotL2, FKBP51, and IQGAP1 in tumor vessels was indicative of a role for these three scaffold proteins in tumor angiogenesis and vascular invasion [56]. They also found a tumor nest enveloped by several telocyte-like FKBP51+ cells. Notably, telocytes are interstitial stromal cells [57] and key players in regenerating and repairing organs. They establish a strict cell-cell interaction within the stem cell *niches* [58] by specific intercellular junctions. Functional interactions between AmotL2, FKBP51, and IQGAP1 proteins in several physiological and pathological situations were confirmed by String analysis showing the involvement of proto-oncogene SRC, HSP90, and yes-associated protein (YAP1) in AmotL2/FKBP51/IQGAP1 multicomplex [5]. Fig. 4 illustrates FKBP51 in the TGF-β signaling.

## FKBP51, Scaffold Roles in Genetic and Epigenetic Regulation

### Histone acetyltransferase (HAT)

**Figure 5:**
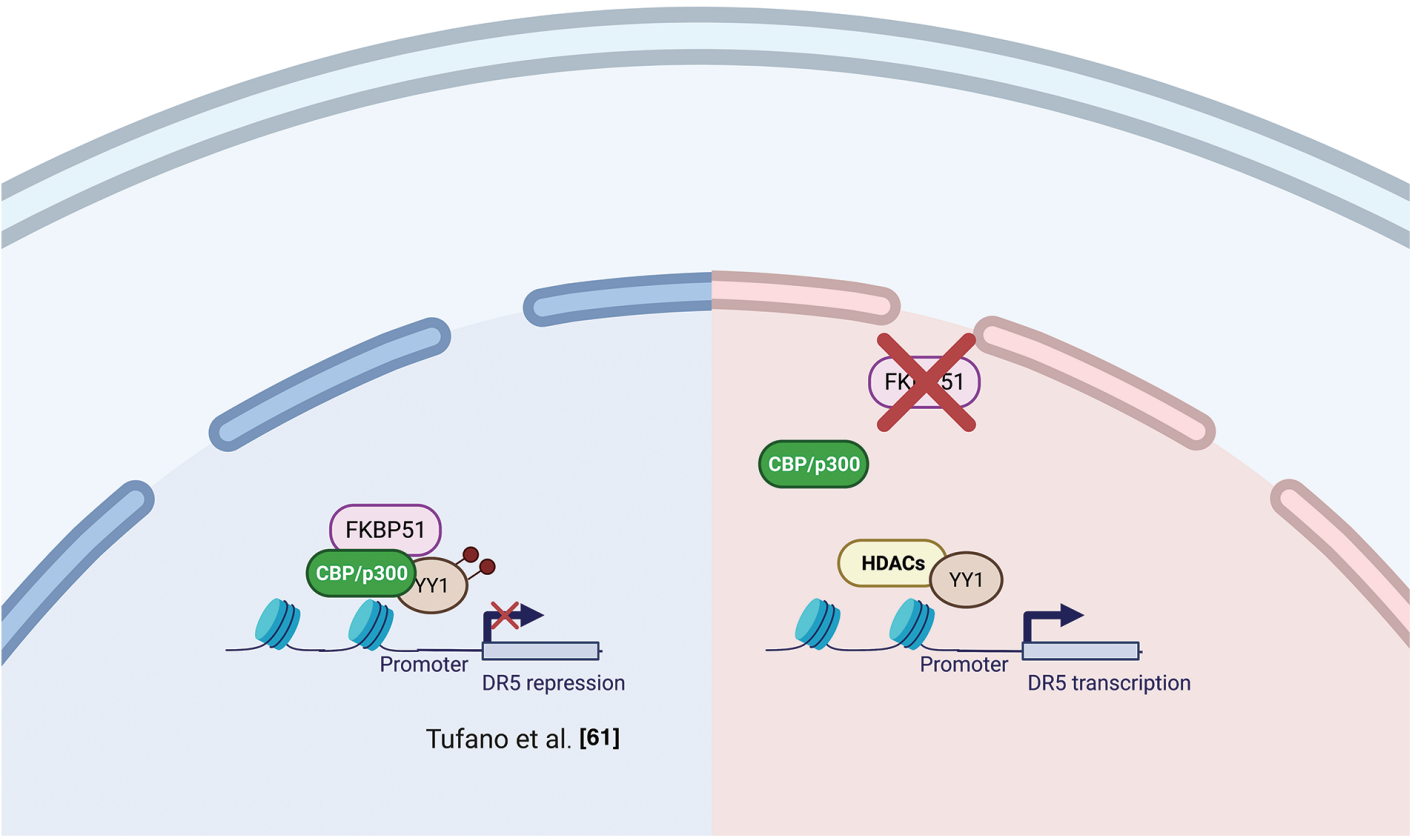
*FKBP51 and p300 and protein acetylation*. Left, FKBP51 interaction with p300/CBP drives to YY1 acetylation and promotes its repressor activity on the DR5 promoter. Right, FKBP51 KO reduces acetyl-YY1 on the DR5 promoter, and DR5 transcript levels increase [61]. The illustration was started from scratch, created with BioRender.com original design.

Several studies support the interaction of FKBP51 with the histone acetyltransferase HAT-p300 [47,54,55]. As mentioned in the AKT section, Yu et al. [47] observed a strict interaction among FKBP51 with p300 and CBP (CREB-binding protein) and found that FKBP51 acetylation was increased by CBP and, to a lesser extent, p300 [47]. *In vitro* assays showed that CBP and p300 acetylated FKBP51, while SIRT7 reverted this post-translational modification [47]. Notably, p300/CBP inhibition is currently explored as anticancer therapy [59,60] as it can affect the transcription of proteins involved in distinct oncogenic networks across different cancer types [59]. The interaction of FKBP51 with p300/CBP suggests an involvement of the immunophilin in chromatin modification [54]. Tufano et al. observed a general increase in HDACs and a reduced level of acetylated-p300 in FKBP51-KO melanoma cells [61]. These KO-cells also showed increased expression of the TNF-related apoptosis-inducing ligand (TRAIL) receptor DR5 and increased sensitivity to TRAIL-induced apoptosis. Death receptor 5 (DR5) expression is inhibited at the transcriptional level by the repressor activity of acetyl-Yin Yang 1 (YY1). YY1 is acetylated by p300 and deacetylated by HDACs [62]. A ChIP assay on KO-cells showed a reduced acetyl-YY1 on the DR5 promoter, associated with increased DR5 transcript levels. Reconstituting FKBP51 levels contrasted the effects of KO on DR5-, acetyl-YY1-, and acetyl-p300-levels [61]. Fig. 5 shows a graphical representation of the proposed relationship between FKBP51 and p300 and protein acetylation.

### CCND1 and Cyclin-dependent kinase (CDK)

**Figure 6:**
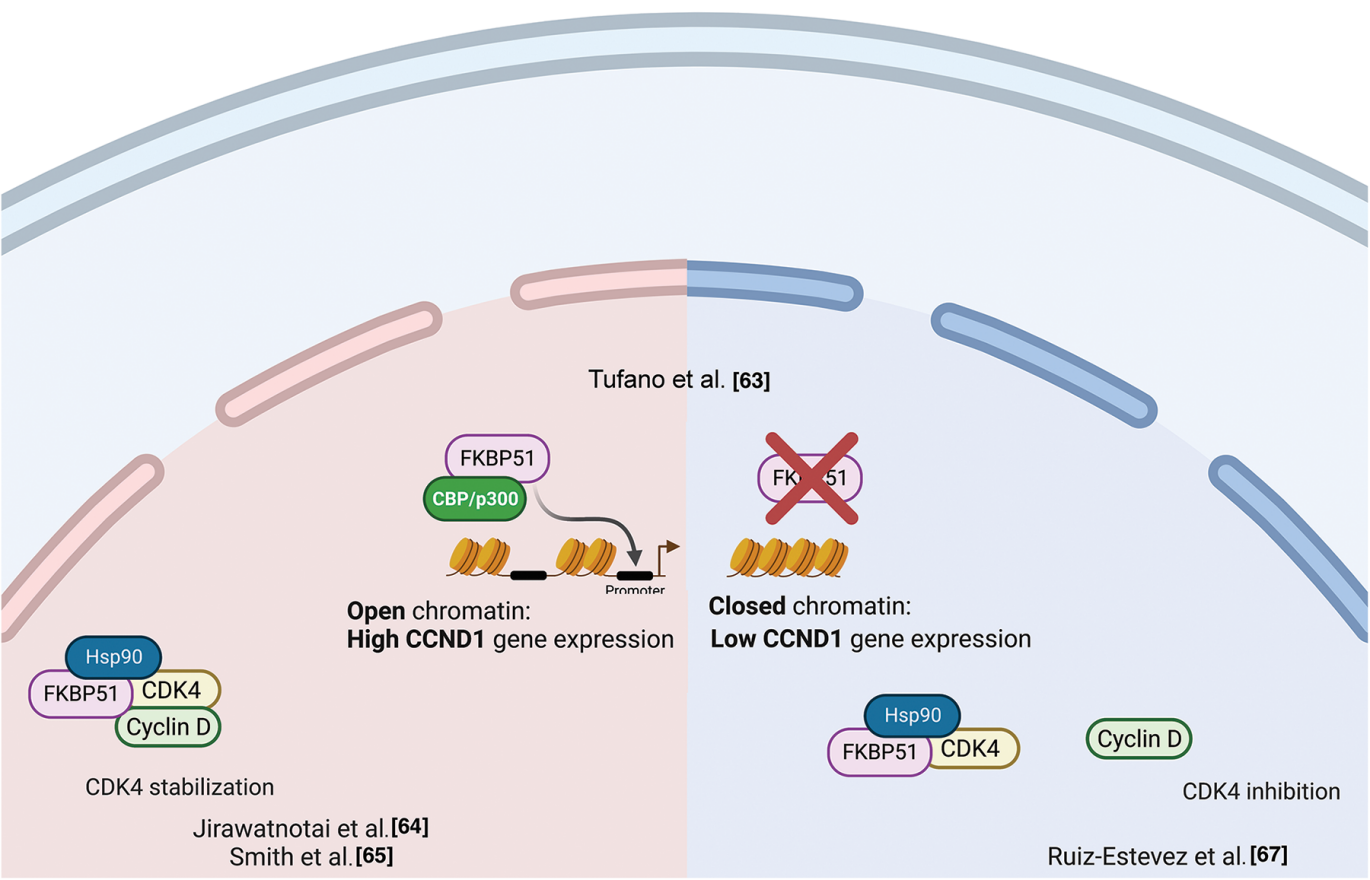
*FKBP51 in cancer cell proliferation and chromatin remodeling*. Left, FKBP51 sequesters CDK4 within the HSP90 storage complex and prevents the formation of the cyclin D1-CDK4 complex [64]. Middle, FKBP51 binds the CCND1 promoter in an open chromatin status accompanied by increased CCND1 transcript levels [63]. Right, FKBP51 interacts and stabilizes CDK4 promoting cell cycle progression and prooncogenic activity [65,66]. The illustration was started from scratch, created with BioRender.com original design.

ChIp studies found FKBP51 bound to the CCND1 gene in an open chromatin status accompanied by increased CCND1 transcript levels [63]. This finding suggested a role for FKBP51 in participating in transcriptional complexes regulating the synthesis of cyclin D. By double immunofluorescence experiments on formalin-fixed and paraffin-embedded CRC samples, Rotoli et al. found FKBP51 and Proliferating cell nuclear antigen (PCNA) protein exhibiting nuclear colocalization in the early S phase in malignant cells forming tumor nests [56]. Cyclin D1 belongs to the core cell cycle machinery. Once induced, it binds and activates the cyclin-dependent kinase (CDK)4 and CDK6. Cyclin D-CDK4/6 holoenzymes phosphorylate proteins, such as the retinoblastoma protein (pRB), which governs cell cycle progression. In breast cancer, Jirawatnotai et al. identified FKBP51 as one of the most abundant CDK4 interacting proteins with a crucial role in CDK4 stability [64]. Newly translated CDK4 is first incorporated into a protein complex containing the chaperone HSP90 and the co-chaperone adaptor CDC37 in the cytoplasm, where CDK4 is stabilized in an inactive form [65,66]. The authors found that CDK4 interacts with FKBP51 or CDC37 but does not form a ternary CDK4-FKBP5-CDC37 complex, suggesting that FKBP51 stabilizes CDK4 level via a CDC37-independent mechanism. The authors found that depletion of FKBP51 decreased CDK4 protein levels and impaired CDK4 kinase activity, suggesting that FKBP51 promotes oncogenesis by stabilizing CDK4. In a mouse study, Ruiz-Estevez et al. [67] confirmed CDK4/FKBP51 interaction, but they found that FKBP51 sequesters CDK4 within the HSP90 storage complex and prevents the formation of the cyclin D1-CDK4 complex. FKBP51, through its isomerase activity, inhibited phosphorylation, hence activating CDK4. By these mechanisms, FKBP51 positively regulated myoblasts differentiation and myogenesis [67]. Fig. 6 illustrates the proposed mechanisms for FKBP51 role in cancer cell proliferation and chromatin remodeling.

### Human telomerase reverse transcriptase (hTERT)

**Figure 7:**
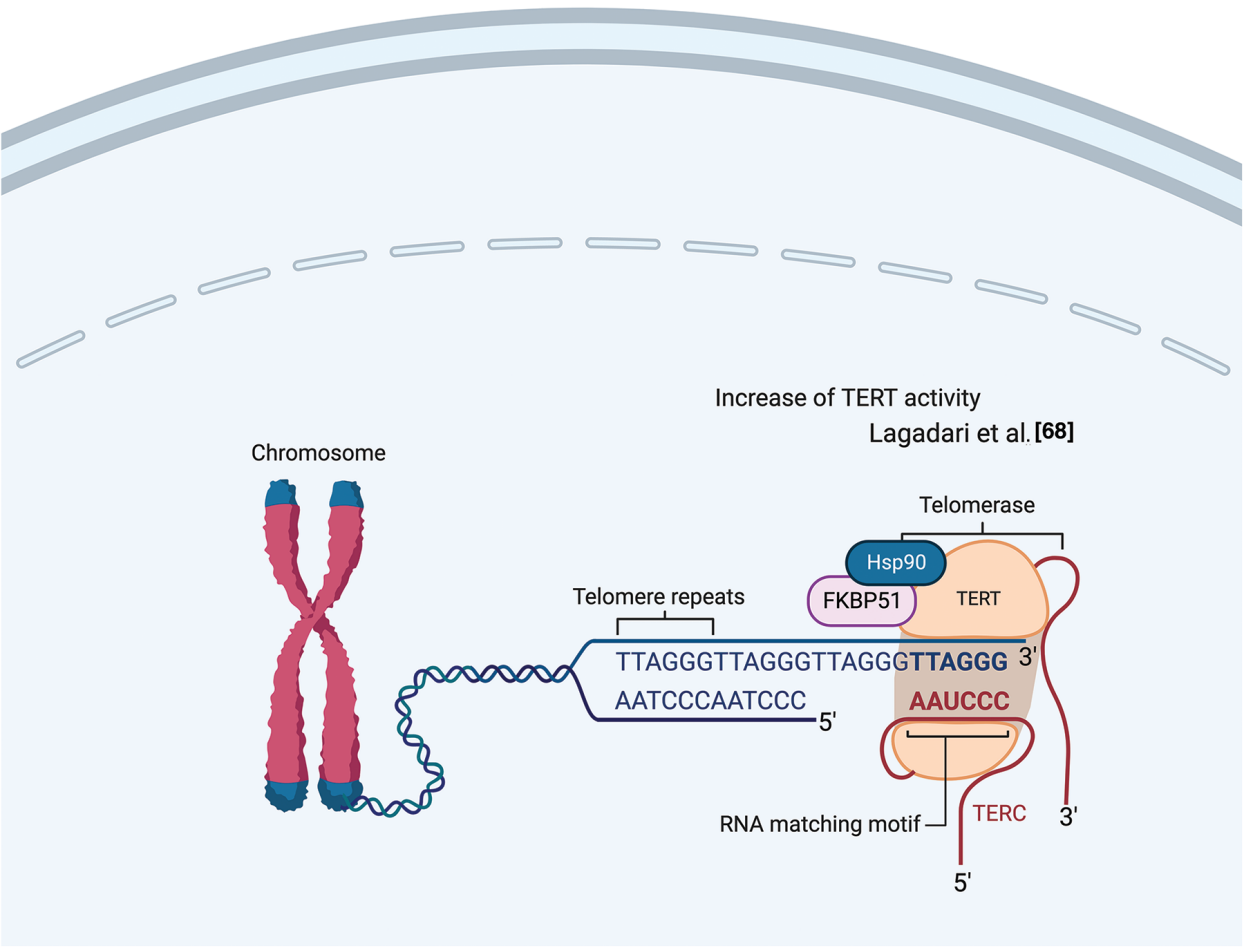
*FKBP51 role in telomerase elongation*. FKBP51 interacts and promotes hTERT catalytic activity into the nucleus [68]. The illustration was started from scratch, created with BioRender.com original design.

Lagadari et al. demonstrated that FKBP51 interacts with human telomerase reverse transcriptase (hTERT) [68]. hTERT is the catalytic subunit of the enzyme telomerase, together with the telomerase RNA component (TERC), which comprises the essential unit of the telomerase complex [68]. As known, telomerase or terminal transferase is a ribonucleoprotein that adds a species-dependent telomere repeat sequence to the 3′ end of telomeres, thus contributing to protecting the end of the chromosome from DNA damage. hTERT is an HSP90 client-protein highly expressed in cancer cells, where it is required to compensate for the loss of telomeric DNA after each successive cell division. Lagadari et al. [68] found that FKBP51 is primarily localized in mitochondria, whereas hTERT is nuclear. Following oxidative stress, FKBP51 becomes nuclear and colocalizes with hTERT. FKBP51 promotes hTERT catalytic activity. The authors identified an essential role for FKBP51 PPIase activity for upregulating hTERT activity and for the TPR domain for efficient interaction of hTERT with HSP90 [68]. Fig. 7 illustrates FKBP51 role in telomerase elongation.

### Argonaute 2 (Ago2)

**Figure 8:**
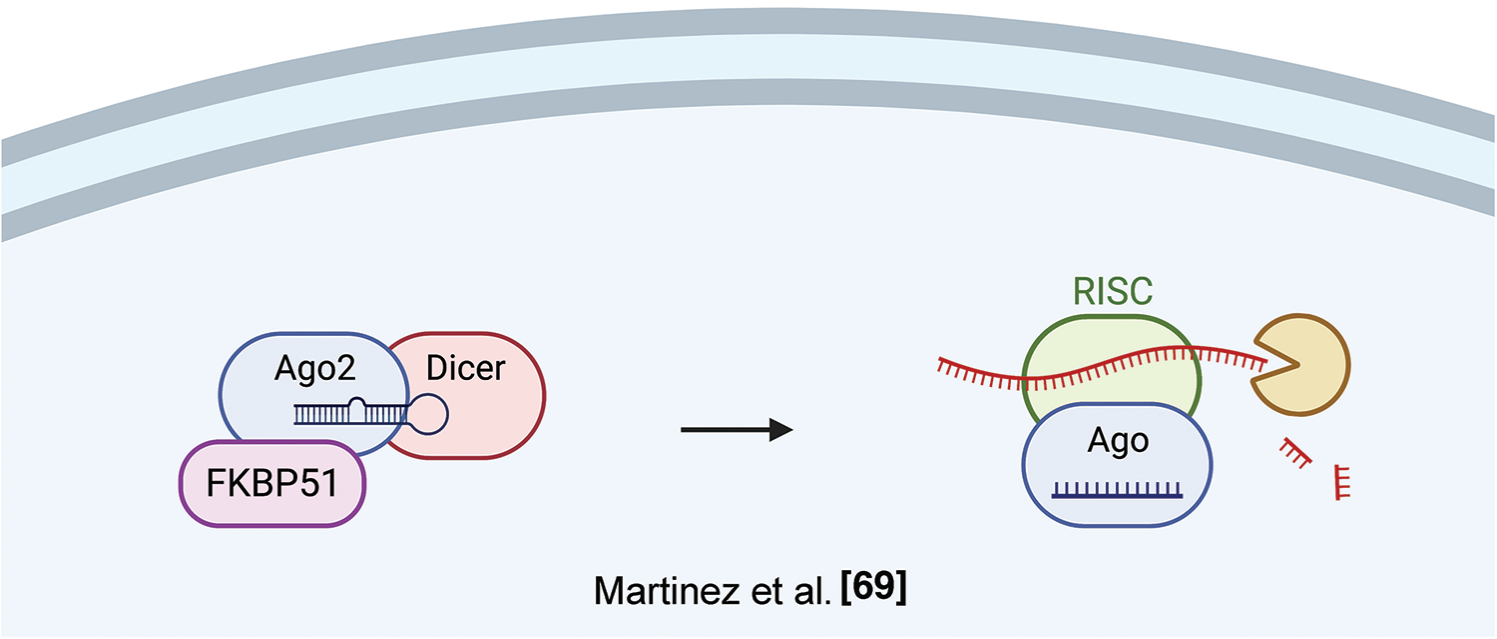
*FKBP51 and RNA processing*. FKBP51 interacts with Ago2 in its RNA-bound structure, thus increasing Ago2 levels and favoring RNA processing [71]. The illustration was started from scratch, created with BioRender.com original design.

A role of FKBP51 in post-transcriptional regulation has been identified by Martinez et al., who found an association of *FKPB5* to Argonaute 2 (Ago 2) in mouse embryonic stem cells (ESCs) [69]. Ago proteins load both siRNAs and miRNAs as short duplexes of ~22 bp; after this event, one strand, the guide, is stably retained while the other strand is degraded. Pharmacological inhibition of the Fkbp5-Ago2 interaction by the immunosuppressant FK506 or by Fkbp5 knockdown blocked miRNA-dependent stabilization of Ago2 expression resulting in decreased Ago2 protein expression, both in mouse and human cells. Ectopic *FKBP5* expression increased Ago2 protein levels in a miRNA-dependent fashion. Given that FK506 binds to active PPIase domain, and FK506 treatment reduced Ago2 levels, along with the presence of two prolines in the RNA-bound Ago2 structure. A crucial role for Fkbp5 in Ago2 regulation was confirmed by mutagenesis analysis, indicating that both the PPIase and TPR domains facilitated Ago1 loading; however, the enzymatic function may be dispensable [70]. Accordingly, another *FKBP, FKBP6*, endowed with an inactive PPIase domain, plays a role in the biogenesis of Piwi-interacting RNAs [71]. Fig. 8 illustrates FKBP51 interaction with Ago2 and RNA processing.

## FKBP51s

### A role as a folder in PDL-1 biogenesis

Among the different protein chaperones that have been reported to either directly or indirectly influence the folding and expression of the programmed death ligand-1 (PDL-1) [72], FKBP51s plays a pivotal role in PDL-1 glycosylation and presentation on the cell surface [73]. PDL-1, also known as CD279 or B7-H1, is a 33-kDa type I transmembrane glycoprotein that contains IgV- and IgC-like domains in its extracellular region [74]. PDL-1 is physiologically expressed in macrophages, some activated T and B cells, dendritic cells (DCs), and some epithelial cells [75,76]. It can be expressed by tumor cells constitutively or adaptively in response to immune cell infiltration [77,78]. Although the molecular mechanism is still poorly characterized, it seems that FKBP51s acts at the level of the endoplasmic reticulum (ER) compartment where PDL-1 precursor protein is synthesized, thereby suggesting a possible role of the chaperone in the biogenesis of PDL-1 [73]. FKBP51s maintains the same structural organization as the full-length FKBP51 except for the lack of the protein-protein interaction domain TPR, which becomes replaced for the alternative splicing by a short new C-terminal amino acidic stretch whose function is still unknown [31]. However, the N-terminal portion of the chaperone consisting of the two PPIase domains (FK1 and FK2) remains intact and, as such, the peptidylprolyl cis/trans isomerase activity [73]. Accordingly, pharmacological inhibition of the PPIase activity by SAFit2 treatment affects PDL-1 glycosylation, plasma membrane expression, and contrasts PDL-1-induced cell death of activated lymphocytes cocultured with glioblastoma cells [73,79]. In an orthotopic mouse model of glioblastoma, daily treatment with SAFit2 significantly reduced tumor PDL-1 expression and growth [79]. Interestingly, PDL-1 belongs to the type I transmembrane protein family. Once the protein is correctly co-translationally translocated and inserted into the ER membrane, its topological organization provides that its proline-rich extracellular portion faces the ER lumen. In contrast, its short C-terminal tail, which does not contain any proline residues, faces the cytosolic side of the ER membrane (UniProt ID: Q9NZQ7). Due to the lack of a needful signal sequence to access the ER lumen, FKBP51s has a cytosolic localization which should not allow either interception or isomerization of proline residues of the PDL-1 polypeptide. D’Arrigo et al. reported that FKBP51s, and not the full-length FKBP51, was enriched in the ER fraction of glioblastoma cell lines suggesting a possible association with the ER membranes [73]. Much evidence has been shown that the ER entry of newly synthesized protein precursors can be assisted by different cytosolic proteins acting either at the level of the Sec61 translocon channel or the associated ribosomes during the co-translational translocation process [80–83]. In *E. coli*, a PPIase-containing chaperone, which plays a crucial role in assisting nascent chains destined for membrane translocation, is the trigger factor (TF) [84–87].

**Figure 9:**
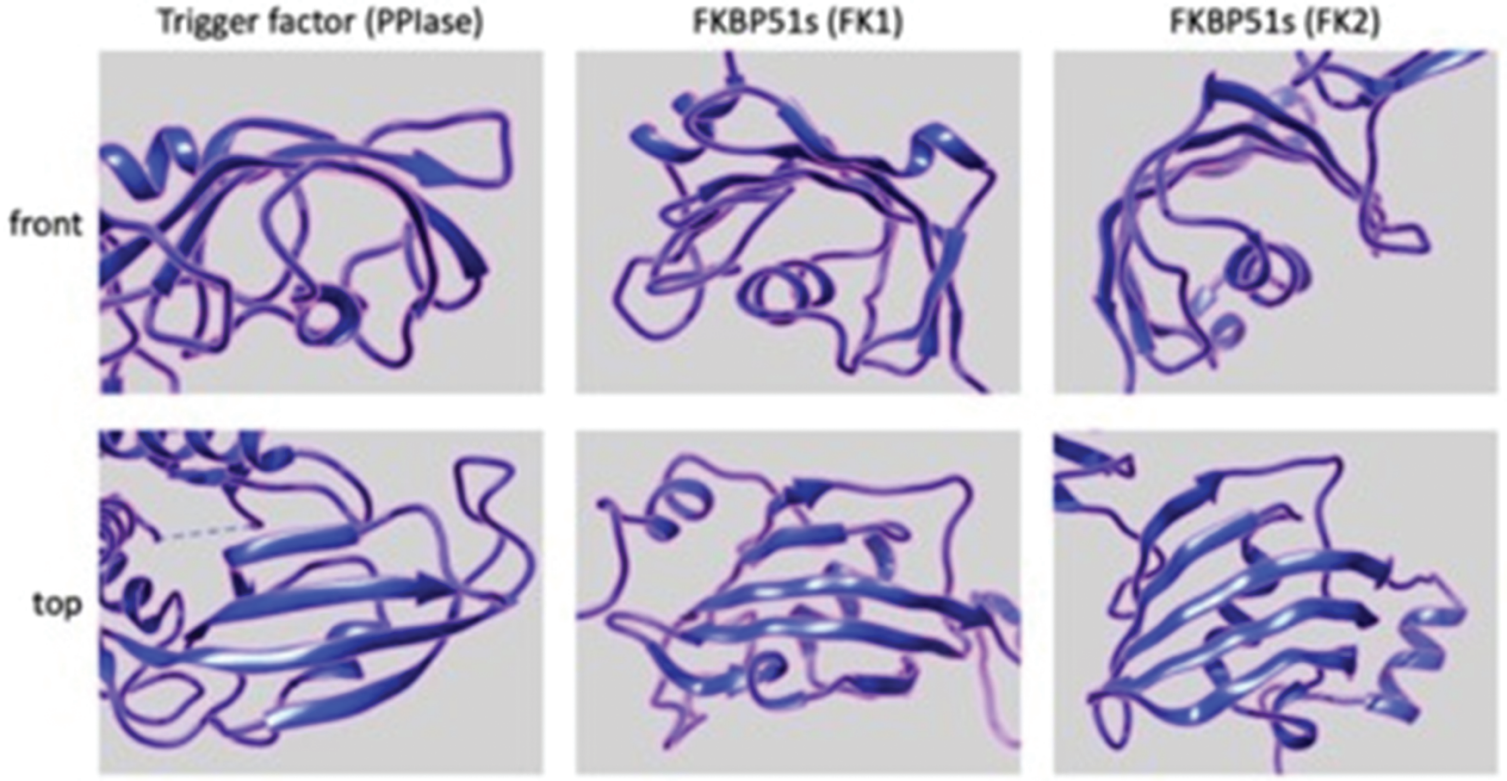
*Putative 3D structures of the PPIse domains*, FK1 (middle panels) and FK2 (right panels), of FKBP51s (https://www.rcsb.org/structure/3O5D) and PPIase domain (left panels) of TF (https://www.rcsb.org/structure/1T11). Front (upper panels) and top (lower panels) views are shown. All the panels have been generated and arranged using the UCSF Chimera software.

Interestingly, the PPIase domains of FKBP51s show somewhat structural similarity to the PPIase domain of TF. Indeed, as shown in Fig. 9, the 3D structure of the PPIase domain belonging to the TF displays a concave shape, which consists mainly of beta sheets with an alpha helix branch inside. This structural organization closely resembles the structure of the two PPIases domains of the FKBP51 protein, suggesting a possible similar function [84–87].

Notably, TF is a specialized chaperone supporting very early the protein folding program [87]. This ribosome-bound protein binds to short nascent chains as they emerge from the ribosome. Around the first 40 amino acids of the nascent chain are protected from the cytosol into the ribosomal exit tunnel [88,89]. Crosslink experiments have shown that TF binds to ribosomal proteins L23 and L29 in the exit tunnel [87]. In particular, the interaction with L23 has been reported as critical [87]. Even if TF possesses PPIase activity, the binding of TF to peptides is independent of the presence of proline residues [90]; therefore, the PPIase activity could not be required for the TF chaperoning of nascent chains.

**Figure 10:**
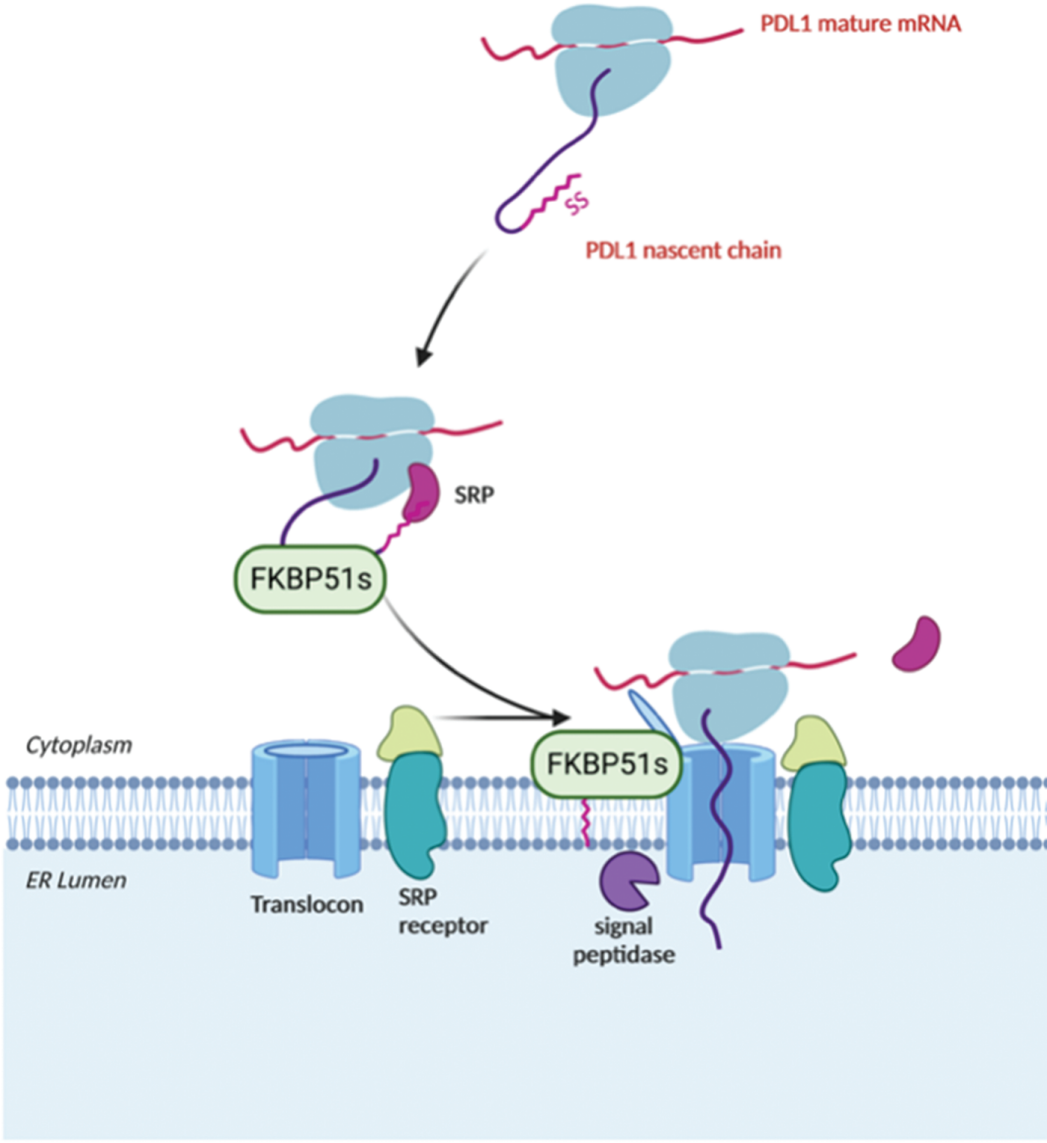
Proposed mechanism for FKBP51s role as PDL-1 foldase FKBP51s may assist PDL-1 nascent chain as it emerges from the ribosome, thereby regulating its biogenesis during the co-translational translocation process of PDL-1 protein into the ER. The illustration was started from scratch, created with BioRender.com original design.

Given the non-luminal ER localization of FKBP51s and the dependence of PDL-1 synthesis on the presence of a functional PPIase domain of FKBP51s, it could be conceivable to speculate that FKBP51s could assist PDL-1’s nascent chain as it emerges from the ribosome, thereby regulating its biogenesis during the co-translational translocation process driving the insertion of PDL-1 protein into the ER membrane. Fig. 10 illustrates the proposed mechanism for FKBP51s role in PDL-1 biogenesis.

### Possible role as a scaffold in transcriptional complex

A recent finding raises the hypothesis that FKBP51s participates, as the canonical FKBP51 in the events that govern cyclin-D oscillation being part of the transcriptional complex bound to CCND1 [63]. Following a study on mouse embryonic fibroblasts (MEFs) demonstrating a role for cyclin-D in the control of PDL-1 expression [91], a study conducted on glioblastoma cell lines showed that the level of PDL-1 changed during the cell cycle [63]. More precisely, PDL-1 mRNA appeared to increase concomitantly to CCND1 on G1/S transition, to decrease during exponential cell growth progressively. In the temporal window of PDL-1 and CCND1 peak, when PDL-1 protein expression level increased on the plasma membrane, FKBP51s localized in ER. On the decrease of cyclin-D and cell proliferation, FKBP51s went nuclear. We interrogated the CCND1 promoter and intronic sequences for their H3K27me3 and H3K4me3 pattern in A375 melanoma FKBP51-KO cells upon exogenous FKBP51 or FKBP51s expression. Our data revealed that H3K4me3 modifications, associated with high transcription activity, occurred in Flag-FKBP51 immunoprecipitated chromatin to the detriment of H3K27me3, thus producing a ratio of α-H3K4me3/α-H3K27me3 >1. In FKBP51s overexpressing cells, ChIp confirmed the binding to CCND1, but the α-H3K4me3/α-H3K27me3 arrangement was consistent with a closed chromatin conformation. In line with this finding, CCND1 mRNA levels were significantly reduced in FKBP51s overexpressing cells, compared to empty vector (EV) levels.

## Conclusions

**Figure 11:**
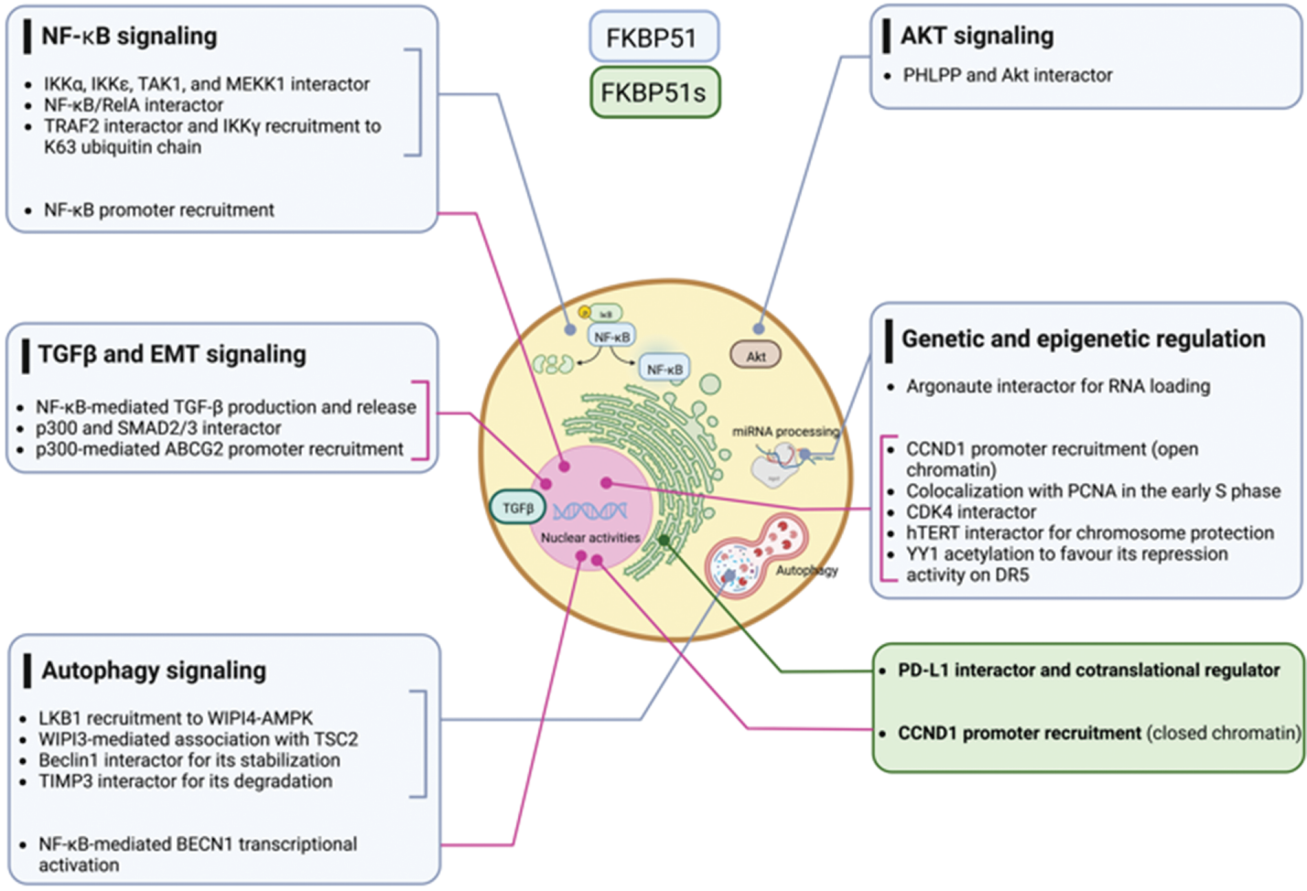
FKBP51 interactions with signaling molecules. The graphical abstract is an overview of the pathways regulated by FKBP5 isoforms in cancer. For details, refer to the text. The illustration was started from scratch, created with BioRender.com original design.

Multiple cancer hallmarks are coordinately modulated in most tumor types by classical oncogenic drivers, including NF-κB, AKT, and TGF-β signaling. Additionally, genetic and epigenetic alterations exert a pivotal role in cancer initiation and progression. The scaffold function of FKBP51 supports these protumoral factors helping in the intracellular assembly of signaling molecules involved. With its splicing isoform, the *FKBP5* gene addresses the tumor requirement to evade the immune system and survive immune attacks. Fig. 11 summarizes FKBP51 interactions and signal transduction pathways examined in this article. Preclinical studies have shown that treatment with drugs that target FKBP5 exerts antitumor effects on glioblastoma mouse models [79,92]. A caveat is, however, envisaged. FKBP51, whether on the one hand is aberrantly expressed by the tumor cells, on the other hand, is constitutively expressed in immune cells with a role in immune activation and proliferation [40]. Thus, as it occurs for many anticancer drugs, FKBP51-targeted therapy can impact the immune system, which deserves careful assessment in clinical trials.

## References

[1] Hu, J., Neiswinger, J., Zhang, J., Zhu, H., Qian, J. (2015). Systematic prediction of scaffold proteins reveals new design principles in scaffold-mediated signal transduction. PLoS Computational Biology*,* 11 *(*9*),* e1004508. 10.1371/journal.pcbi.1004508; 26393507 PMC4578958

[2] Faux, M. C., Scott, J. D. (1996). Molecular glue: Kinase anchoring and scaffold proteins. Cell*,* 85 *(*1*),* 9–12. 10.1016/S0092-8674(00)81075-2; 8620541

[3] Garbett, D., Bretscher, A. (2014). The surprising dynamics of scaffolding proteins. Molecular Biology Cell*,* 25 *(*16*),* 2315–2319. 10.1091/mbc.e14-04-0878; 25122925 PMC4142605

[4] Li, C., Wang, H., Yao, H., Fang, J. Y., Xu, J. (2017). Scaffold proteins in gastrointestinal tumors as a shortcut to oncoprotein activation. Gastrointestinal Tumors*,* 4 *(*1–2*),* 1–10. 10.1159/000477904; 29071259 PMC5649239

[5] White, C. D., Brown, M. D., Sacks, D. B. (2009). IQGAPs in cancer: A family of scaffold proteins underlying tumorigenesis. FEBS Letters*,* 583 *(*12*),* 1817–1824. 10.1016/j.febslet.2009.05.007; 19433088 PMC2743239

[6] Tornillo, G., Defilippi, P., Cabodi, S. (2014). Cas proteins: Dodgy scaffolding in breast cancer. Breast Cancer Research*,* 16 *(*5*),* 443. 10.1186/s13058-014-0443-5; 25606587 PMC4384296

[7] Shagisultanova, E., Gaponova, A. V., Gabbasov, R., Nicolas, E., Golemis, E. A. (2015). Preclinical and clinical studies of the NEDD9 scaffold protein in cancer and other diseases. Gene*,* 567 *(*1*),* 1–11. 10.1016/j.gene.2015.04.086; 25967390 PMC4458429

[8] Kawaguchi, K., Asano, S. (2022). Pathophysiological roles of actin-binding scaffold protein, Ezrin. International Journal of Molecular Science*,* 23 *(*6*),* 3246. 10.3390/ijms23063246; 35328667 PMC8952289

[9] Tibbe, D., Ferle, P., Krisp, C., Nampoothiri, S., Mirzaa, G. et al. (2022). Regulation of Liprin-α phase separation by CASK is disrupted by a mutation in its CaM kinase domain. Life Science Alliance*,* 5 *(*10*),* e202201512. 10.26508/lsa.202201512; 36137748 PMC9500383

[10] Li, O., Alsaidan, O. A., Ma, Y., Kim, S., Liu, J. et al. (2018). Pharmacologically targeting the myristoylation of the scaffold protein FRS2α inhibits FGF/FGFR-mediated oncogenic signaling and tumor progression. Journal of Biological Chemistry*,* 293 *(*17*),* 6434–6448. 10.1074/jbc.RA117.000940; 29540482 PMC5925806

[11] Yang, M., Li, C., Li, Y., Cheng, C., Shi, M. et al. (2022). Design, synthesis, biological evaluation and molecular docking study of 2,4-diarylimidazoles and 2,4-bis(benzyloxy)-5-arylpyrimidines as novel HSP90 N-terminal inhibitors. Journal of Enzyme Inhibition and Medical Chemistry*,* 37 *(*1*),* 2551–2565. 10.1080/14756366.2022.2124407; 36120957 PMC9518286

[12] Finger, E. C., Castellini, L., Rankin, E. B., Vilalta, M., Krieg, A. J. et al. (2015). Hypoxic induction of AKAP12 variant 2 shifts PKA-mediated protein phosphorylation to enhance migration and metastasis of melanoma cells. Proceeding of the National Academy of Sciences of United States of America*,* 112 *(*14*),* 4441–4446. 10.1073/pnas.1418164112; 25792458 PMC4394282

[13] Maiques-Diaz, A., Nicosia, L., Basma, N. J., Romero-Camarero, I., Camera, F. et al. (2022). HMG20B stabilizes association of LSD1 with GFI1 on chromatin to confer transcription repression and leukemia cell differentiation block. Oncogene*,* 41 *(*44*),* 4841–4854. 10.1038/s41388-022-02471-y; 36171271 PMC7613766

[14] Toker, A. (2012). Achieving specificity in Akt signaling in cancer. Advances in Biological Regulation*,* 52 *(*1*),* 78–87. 10.1016/j.advenzreg.2011.09.020; 21986444 PMC3614006

[15] Bao, F., Hao, P., An, S., Yang, Y., Liu, Y. et al. (2021). Akt scaffold proteins: The key to controlling specificity of Akt signaling. American Journal of Physiology-Cell Physiology*,* 321 *(*3*),* C429–C442. 10.1152/ajpcell.00146.2020; 34161152

[16] Bellizzi, A., Greco, M. R., Rubino, R., Paradiso, A., Forciniti, S. et al. (2015). The scaffolding protein NHERF1 sensitizes EGFR-dependent tumor growth, motility and invadopodia function to gefitinib treatment in breast cancer cells. International Journal of Oncology*,* 46 *(*3*),* 1214–1224. 10.3892/ijo.2014.2805; 25530180

[17] Fischer, G., Aumüller, T. (2003). Regulation of peptide bond cis/trans isomerization by enzyme catalysis and its implication in physiological processes. Reviews of Physiology, Biochemistry and Pharmacology*,* 148*,* 105–150. 10.1007/978-3-540-44834-1 12698322

[18] Dornan, J., Taylor, P., Walkinshaw, M. D. (2003). Structures of immunophilins and their ligand complexes. Current Topics in Medical Chemistry*,* 3 *(*12*),* 1392–1409. 10.2174/1568026033451899; 12871171

[19] Rein, T. (2020). Peptidylprolylisomerases, protein folders, or scaffolders? The example of FKBP51 and FKBP52. BioEssays*,* 42 *(*7*),* e1900250. 10.1002/bies.201900250; 32323357

[20] Barik, S. (2006). Immunophilins: For the love of proteins. Cellular and Molecular Life Sciences CMLS*,* 63 *(*24*),* 2889–2900. 10.1007/s00018-006-6215-3; 17075696 PMC11136219

[21] Nigro, P., Pompilio, G., Capogrossi, M. C. (2013). Cyclophilin A: A key player for human disease. Cell Death & Disease*,* 4 *(*10*),* e888. 10.1038/cddis.2013.410; 24176846 PMC3920964

[22] Lee, J., Kim, S. S. (2010). An overview of cyclophilins in human cancers. Journal of International Medical Research*,* 38 *(*5*),* 1561–1574. 10.1177/147323001003800501; 21309470

[23] Villmow, M., Baumann, M., Malesevic, M., Sachs, R., Hause, G. et al. (2016). Inhibition of Aβ(1-40) fibril formation by cyclophilins. Biochemical Journal*,* 473 *(*10*),* 1355–1368. 10.1042/BCJ20160098; 26994210

[24] Schiene-Fischer, C. (2015). Multidomain peptidyl prolyl cis/trans isomerases. Biochimica et Biophysica Acta (BBA)—General Subjects*,* 850 *(*10*),* 2005–2016. 10.1016/j.bbagen.2014.11.012; 25445709

[25] Lavin, P. T., Mc Gee, M. M. (2015). Cyclophilin function in cancer; Lessons from virus replication. Current Molecular Pharmacology*,* 9 *(*2*),* 148–164. 10.2174/1874467208666150519115443; 25986562

[26] Peterson, A. A., Rangwala, A. M., Thakur, M. K., Ward, P. S., Hung, C. et al. (2022). Discovery and molecular basis of subtype-selective cyclophilin inhibitors. Nature Chemical Biology*,* 18 *(*11*),* 1184–1195. 10.1038/s41589-022-01116-1; 36163383 PMC9596378

[27] Romano, S., D’Angelillo, A., Romano, M. F. (2015). Pleiotropic roles in cancer biology for multifaceted proteins FKBPs. Biochimica et Biophysica Acta (BBA)—General Subjects*,* 1850 *(*10*),* 2061–2068. 10.1016/j.bbagen.2015.01.004; 25592270

[28] Lv, S., Zhao, X., Zhang, E., Yan, Y., Ma, X. et al. (2022). Lysine demethylase KDM1A promotes cell growth via FKBP8-BCL2 axis in hepatocellular carcinoma. The Journal of Biological Chemistry*,* 298 *(*9*),* 102374. 10.1016/j.jbc.2022.102374; 35970393 PMC9478407

[29] Huang, J., Zhao, Y. (2022). E2F transcription factor 1 activates FKBP prolyl isomerase 4 to promote angiogenesis in cervical squamous cell carcinoma via the PI3K/AKT signaling pathway. Reproductive Science*,* 30 *(*4*),* 1229–1240. 10.1007/s43032-022-01034-6; 35849266

[30] Zhu, Z., Hou, Q., Wang, B., Li, C., Liu, L. et al. (2022). FKBP4 regulates 5-fluorouracil sensitivity in colon cancer by controlling mitochondrial respiration. Life Science Alliance*,* 5 *(*11*),* e202201413. 10.26508/lsa.202201413; 35981890 PMC9389594

[31] Romano, S., D’Angelillo, A., Staibano, S., Simeone, E., D’Arrigo, P. et al. (2015). Immunomodulatory pathways regulate expression of a spliced FKBP51 isoform in lymphocytes of melanoma patients. Pigment Cell & Melanoma Research*,* 28 *(*4*),* 442–452. 10.1111/pcmr.12378; 25895097

[32] Sinars, C. R., Cheung-Flynn, J., Rimerman, R. A., Scammell, J. G., Smith, D. F. et al. (2003). Structure of the large FK506-binding protein FKBP51, an Hsp90-binding protein and a component of steroid receptor complexes. Proceeding of the Nationall Academy of sciences of United States of America*,* 100 *(*3*),* 868–873. 10.1073/pnas.0231020100; 12538866 PMC298693

[33] Bracher, A., Kozany, C., Thost, A. K., Hausch, F. (2011). Structural characterization of the PPIase domain of FKBP51, a cochaperone of human Hsp90. Acta Crystallographica, Section D, Biological Crystallography*,* 67*,* 549–559. 10.1107/S0907444911013862; 21636895

[34] David, C. J., Manley, J. L. (2010). Alternative pre-mRNA splicing regulation in cancer: pathways and programs unhinged. Genes & Development*,* 24 *(*21*),* 2343–2364. 10.1101/gad.1973010; 21041405 PMC2964746

[35] Hähle, A., Merz, S., Meyners, C., Hausch, F. (2019). The many faces of FKBP51. Biomolecules*,* 9 *(*1*),* 35. 10.3390/biom9010035; 30669684 PMC6359276

[36] Vittorioso, P., Cowling, R., Faure, J. D., Caboche, M., Bellini, C. (1998). Mutation in the Arabidopsis PASTICCINO1 gene, which encodes a new FK506-binding protein-like protein, has a dramatic effect on plant development. Molecular and Cellular Biology*,* 18 *(*5*),* 3034–3043. 10.1128/MCB.18.5.3034; 9566922 PMC110682

[37] Yeh, W. C., Li, T. K., Bierer, B. E., Yang, W. M. (1995). Identification and characterization of an immunophilin expressed during the clonal expansion phase of adipocyte differentiation. Proceeding of the Nationall Academy of sciences of United States of America*,* 92 *(*24*),* 11081–11085. 10.1073/pnas.92.24.11081; 7479941 PMC40575

[38] Liu, T. M., Martina, M., Hutmacher, D. W., Hui, J. H., Lee, E. H. et al. (2007). Identification of common pathways mediating differentiation of bone marrow- and adipose tissue-derived human mesenchymal stem cells into three mesenchymal lineages. Stem Cells*,* 25 *(*3*),* 750–760. 10.1634/stemcells.2006-0394; 17095706

[39] Smedlund, K. B., Sanchez, E. R., Hinds Jr, T. D. (2021). FKBP51 and the molecular chaperoning of metabolism. Trends in Endocrinoly and Metabolism*,* 32 *(*11*),* 862–874. 10.1016/j.tem.2021.08.003; 34481731 PMC8516732

[40] Marrone, L., D’Agostino, M., Cesaro, E., di Giacomo, V., Urzini, S. et al. (2023). Alternative splicing of *FKBP5* gene exerts control over T lymphocyte expansion. Journal of Cellular Biochemistry. 10.1002/jcb.30364; 36645880

[41] Bouwmeester, T., Bauch, A., Ruffner, H., Angrand, P. O., Bergamini, G. et al. (2004). A physical and functional map of the human TNF-α/NF-κB signal transduction pathway. Nature Cell Biology*,* 6 *(*2*),* 97–105. 10.1038/ncb1086; 14743216

[42] Erlejman, A. G., de Leo, S. A., Mazaira, G. I., Molinari, A. M., Camisay, M. F. et al. (2014). NF-κB transcriptional activity is modulated by FK506-binding proteins FKBP51 and FKBP52: A role for peptidyl-prolyl isomerase activity. The Journal of Biological Chemistry*,* 289 *(*38*),* 26263–26276. 10.1074/jbc.M114.582882; 25104352 PMC4176250

[43] Romano, S., Xiao, Y., Nakaya, M., D’Angelillo, A., Chang, M. et al. (2015). FKBP51 employs both scaffold and isomerase functions to promote NF-κB activation in melanoma. Nucleic Acids Research*,* 43 *(*14*),* 6983–6993. 10.1093/nar/gkv615; 26101251 PMC4538817

[44] Romano, S., Tufano, M., D’Arrigo, P., Vigorito, V., Russo, S. et al. (2020). Cell stemness, epithelial-to-mesenchymal transition, and immunoevasion: Intertwined aspects in cancer metastasis. Seminars Cancer Biology*,* 60 *(*70*),* 181–190. 10.1016/j.semcancer.2019.08.015; 31422157

[45] Pei, H., Li, L., Fridley, B. L., Jenkins, G. D., Kalari, K. R. et al. (2009). FKBP51 affects cancer cell response to chemotherapy by negatively regulating Akt. Cancer Cell*,* 16 *(*3*),* 259–266. 10.1016/j.ccr.2009.07.016; 19732725 PMC2755578

[46] Fabian, A. K., März, A., Neimanis, S., Biondi, R. M., Kozany, C. et al. (2013). InterAKTions with FKBPs--mutational and pharmacological exploration. PLoS One*,* 8 *(*2*),* e57508. 10.1371/journal.pone.0057508; 23469007 PMC3585324

[47] Yu, J., Qin, B., Wu, F., Qin, S., Nowsheen, S. et al. (2017). Regulation of serine-threonine kinase akt activation by NAD+-dependent deacetylase SIRT7. Cell Reports*,* 18 *(*5*),* 1229–1240. 10.1016/j.celrep.2017.01.009; 28147277 PMC5298804

[48] Gassen, N. C., Hartmann, J., Schmidt, M. V., Rein, T. (2015). FKBP5/FKBP51 enhances autophagy to synergize with antidepressant action. Autophagy*,* 11 *(*3*),* 578–580. 10.1080/15548627.2015.1017224; 25714272 PMC4502647

[49] Tufano, M., Marrone, L., D’Ambrosio, C., di Giacomo, V., Urzini, S. et al. (2023). FKBP51 plays an essential role in Akt ubiquitination that requires Hsp90 and PHLPP. Cell Death & Disease*,* 14 *(*2*)*. 10.1038/s41419-023-05629-y PMC9925821 36781840

[50] Häusl, A. S., Bajaj, T., Brix, L. M., Pöhlmann, M. L., Hafner, K. et al. (2022). Mediobasal hypothalamic FKBP51 acts as a molecular switch linking autophagy to whole-body metabolism. Science Advances*,* 8 *(*10*),* eabi4797. 10.1126/sciadv.abi4797; 35263141 PMC8906734

[51] Bakula, D., Müller, A., Zuleger, T., Takacs, Z., Franz-Wachtel, M. et al. (2017). WIPI3 and WIPI4 β-propellers are scaffolds for LKB1-AMPK-TSC signalling circuits in the control of autophagy. Nature Communication*,* 8 *(*1*),* 15637. 10.1038/ncomms15637; 28561066 PMC5460038

[52] Giraudier, S., Chagraoui, H., Komura, E., Barnache, S., Blanchet, B. et al. (2002). Overexpression of FKBP51 in idiopathic myelofibrosis regulates the growth factor independence of megakaryocyte progenitors. Blood*,* 100 *(*8*),* 2932–2940. 10.1182/blood-2002-02-0485; 12351405

[53] Komura, E., Tonetti, C., Penard-Lacronique, V., Chagraoui, H., Lacout, C. et al. (2005). Role for the nuclear factor κB pathway in transforming growth factor-beta1 production in idiopathic myelofibrosis: Possible relationship with FK506 binding protein 51 overexpression. Cancer Research*,* 65 *(*8*),* 3281–3289. 10.1158/0008-5472.CAN-04-2339; 15833861

[54] Romano, S., Staibano, S., Greco, A., Brunetti, A., Nappo, G. et al. (2013). FK506 binding protein 51 positively regulates melanoma stemness and metastatic potential. Cell Death and Disease*,* 4 *(*4*),* e578. 10.1038/cddis.2013.109; 23559012 PMC3641332

[55] Romano, S., D’Angelillo, A., D’Arrigo, P., Staibano, S., Greco, A. et al. (2014). FKBP51 increases the tumour-promoter potential of TGF-beta. Clinical and Translational Medicine*,* 3 *(*1*),* 1. 10.1186/2001-1326-3-1; 24460977 PMC3906759

[56] Rotoli, D., Morales, M., Ávila, J., Maeso, M. D. C., García, M. D. P. et al. (2017). Commitment of scaffold proteins in the onco-biology of human colorectal cancer and liver metastases after oxaliplatin-based chemotherapy. International Journal of Molecular Sciences*,* 18 *(*4*),* 891. 10.3390/ijms18040891; 28441737 PMC5412470

[57] Popescu, L. M., Faussone-Pellegrini, M. S. (2010). TELOCYTES—a case of serendipity: The winding way from Interstitial Cells of Cajal (ICC), via Interstitial Cajal-Like Cells (ICLC) to TELOCYTES. Journal of Cellular and Molecular Medicine*,* 14 *(*4*),* 729–740. 10.1111/j.1582-4934.2010.01059.x; 20367664 PMC3823108

[58] Polykandriotis, E., Popescu, L. M., Horch, R. E. (2010). Regenerative medicine: Then and now--An update of recent history into future possibilities. Journal of Cellular and Molecular Medicine*,* 14 *(*10*),* 2350–2358. 10.1111/j.1582-4934.2010.01169.x; 20825521 PMC3823153

[59] Hogg, S. J., Motorna, O., Cluse, L. A., Johanson, T. M., Coughlan, H. D. et al. (2021). Targeting histone acetylation dynamics and oncogenic transcription by catalytic P300/CBP inhibition. Molecular Cell*,* 81 *(*10*),* 2183–2200.e13. 10.1016/j.molcel.2021.04.015; 34019788 PMC8183601

[60] Spriano, F., Gaudio, E., Cascione, L., Tarantelli, C., Melle, F. et al. (2020). Antitumor activity of the dual BET and CBP/EP300 inhibitor NEO2734. Blood Advances*,* 4 *(*17*),* 4124–4135. 10.1182/bloodadvances.2020001879; 32882003 PMC7479962

[61] Tufano, M., Cesaro, E., Martinelli, R., Pacelli, R., Romano, S. et al. (2021). FKBP51 affects TNF-related apoptosis inducing ligand response in melanoma. Frontiers in Cell and Developmental Biology*,* 9*,* 718947. 10.3389/fcell.2021.718947; 34589486 PMC8473884

[62] Yao, Y. L., Yang, W. M., Seto, E. (2001). Regulation of transcription factor YY1 by acetylation and deacetylation. Molecular and Cellular Biology*,* 21 *(*17*),* 5979–5991. 10.1128/MCB.21.17.5979-5991.2001; 11486036 PMC87316

[63] Tufano, M., D’Arrigo, P., D’Agostino, M., Giordano, C., Marrone, L. et al. (2021). PD-L1 expression fluctuates concurrently with cyclin D in glioblastoma cells. Cells*,* 10 *(*9*),* 2366. 10.3390/cells10092366; 34572014 PMC8468141

[64] Jirawatnotai, S., Sharma, S., Michowski, W., Suktitipat, B., Geng, Y. et al. (2014). The cyclin D1-CDK4 oncogenic interactome enables identification of potential novel oncogenes and clinical prognosis. Cell Cycle*,* 13 *(*18*),* 2889–2900. 10.4161/15384101.2014.946850; 25486477 PMC4614005

[65] Smith, J. R., de Billy, E., Hobbs, S., Powers, M., Prodromou, C. et al. (2015). Restricting direct interaction of CDC37 with HSP90 does not compromise chaperoning of client proteins. Oncogene*,* 34 *(*1*),* 15–26. 10.1038/onc.2013.519; 24292678 PMC3984902

[66] Vaughan, C. K., Mollapour, M., Smith, J. R., Truman, A., Hu, B. et al. (2008). Hsp90-dependent activation of protein kinases is regulated by chaperone-targeted dephosphorylation of Cdc37. Molecular Cell*,* 31 *(*6*),* 886–895. 10.1016/j.molcel.2008.07.021; 18922470 PMC2568865

[67] Ruiz-Estevez, M., Staats, J., Paatela, E., Munson, D., Katoku-Kikyo, N. et al. (2018). Promotion of myoblast differentiation by Fkbp5 via Cdk4 isomerization. Cell Reports*,* 25 *(*9*),* 2537–2551.e8. 10.1016/j.celrep.2018.11.006; 30485818 PMC6350781

[68] Lagadari, M., Zgajnar, N. R., Gallo, L. I., Galigniana, M. D. (2016). Hsp90-binding immunophilin FKBP51 forms complexes with hTERT enhancing telomerase activity. Molecular Oncology*,* 10 *(*7*),* 1086–1098. 10.1016/j.molonc.2016.05.002; 27233944 PMC5423183

[69] Martinez, N. J., Chang, H. M., Borrajo Jde, R., Gregory, R. I. (2013). The co-chaperones Fkbp4/5 control Argonaute2 expression and facilitate RISC assembly. RNA*,* 19 *(*11*),* 1583–1593. 10.1261/rna.040790.113; 24049110 PMC3851725

[70] Iki, T., Yoshikawa, M., Meshi, T., Ishikawa, M. (2012). Cyclophilin 40 facilitates HSP90-mediated RISC assembly in plants. The EMBO Journal*,* 31 *(*2*),* 267–278. 10.1038/emboj.2011.395; 22045333 PMC3261558

[71] Olivieri, D., Senti, K. A., Subramanian, S., Sachidanandam, R., Brennecke, J. (2012). The cochaperone shutdown defines a group of biogenesis factors essential for all piRNA populations in Drosophila. Molecular Cell*,* 47 *(*6*),* 954–969. 10.1016/j.molcel.2012.07.021; 22902557 PMC3463805

[72] De Almeida, S., Regimbeau, M., Jego, G., Garrido, C., Girodon, F. et al. (2020). Heat shock proteins and PD-1/PD-L1 as potential therapeutic targets in myeloproliferative neoplasms. Cancers*,* 12 *(*9*),* 2592. 10.3390/cancers12092592; 32932806 PMC7563255

[73] D’Arrigo, P., Russo, M., Rea, A., Tufano, M., Guadagno, E. et al. (2017). A regulatory role for the co-chaperone FKBP51s in PD-L1 expression in glioma. Oncotarget*,* 8 *(*40*),* 68291–68304. 10.18632/oncotarget.19309; 28978117 PMC5620257

[74] Youngnak, P., Kozono, Y., Kozono, H., Iwai, H., Otsuki, N. et al. (2003). Differential binding properties of B7-H1 and B7-DC to programmed death-1. Biochemical and Biophysical Research Communications*,* 307 *(*3*),* 672–677. 10.1016/S0006-291X(03)01257-9; 12893276

[75] Yamazaki, T., Akiba, H., Iwai, H., Matsuda, H., Aoki, M. et al. (2002). Expression of programmed death 1 ligands by murine T cells and APC. Journal of Immunology*,* 169 *(*10*),* 5538–5545. 10.4049/jimmunol.169.10.5538; 12421930

[76] Keir, M. E., Butte, M. J., Freeman, G. J., Sharpe, A. H. (2008). PD-1 and its ligands in tolerance and immunity. Annual Review of Immunology*,* 26 *(*1*),* 677–704. 10.1146/annurev.immunol.26.021607.090331; 18173375 PMC10637733

[77] Freeman, G. J., Long, A. J., Iwai, Y., Bourque, K., Chernova, T. et al. (2000). Engagement of the PD-1 immunoinhibitory receptor by a novel B7 family member leads to negative regulation of lymphocyte activation. The Journal of Experimental Medicine*,* 192 *(*7*),* 1027–1034. 10.1084/jem.192.7.1027; 11015443 PMC2193311

[78] Diskin, B., Adam, S., Cassini, M. F., Sanchez, G., Liria, M. et al. (2020). PD-L1 engagement on T cells promotes self-tolerance and suppression of neighboring macrophages and effector T cells in cancer. Nature Immunology*,* 21 *(*4*),* 442–454. 10.1038/s41590-020-0620-x; 32152508

[79] D’Arrigo, P., Digregorio, M., Romano, S., Tufano, M., Rea, A. et al. (2019). The splicing FK506-binding protein-51 isoform plays a role in glioblastoma resistance through programmed cell death ligand-1 expression regulation. Cell Death Discovery*,* 5 *(*1*),* 137. 10.1038/s41420-019-0216-0; 31583120 PMC6760221

[80] Craig, E. A. (2018). Hsp70 at the membrane: Driving protein translocation. BMC Biology*,* 16 *(*1*),* 11. 10.1186/s12915-017-0474-3; 29343244 PMC5773037

[81] Larburu, N., Adams, C. J., Chen, C. S., Nowak, P. R., Ali, M. M. U. (2021). Mechanism of Hsp70 specialized interactions in protein translocation and the unfolded protein response. Open Biology*,* 10 *(*8*),* 200089. 10.1098/rsob.200089; 32810420 PMC7479934

[82] Kriegler, T., Kiburg, G., Hessa, T. (2020). Translocon-Associated Protein Complex (TRAP) is crucial for co-translational translocation of pre-proinsulin. Journal of Molecular Biology*,* 432 *(*24*),* 166694. 10.1016/j.jmb.2020.10.028; 33137310

[83] Hsieh, H. H., Lee, J. H., Chandrasekar, S., Shan, S. O. (2020). A ribosome-associated chaperone enables substrate triage in a cotranslational protein targeting complex. Nature Communications*,* 11 *(*1*),* 5840. 10.1038/s41467-020-19548-5; 33203865 PMC7673040

[84] Stoller, G., Rücknagel, K. P., Nierhaus, K. H., Schmid, F. X., Fischer, G. et al. (1995). A ribosome-associated peptidyl-prolyl cis/trans isomerase identified as the trigger factor. The EMBO Journal*,* 14 *(*20*),* 4939–4948. 10.1002/j.1460-2075.1995.tb00177.x; 7588623 PMC394597

[85] Bornemann, T., Holtkamp, W., Wintermeyer, W. (2014). Interplay between trigger factor and other protein biogenesis factors on the ribosome. Nature Communications*,* 5 *(*1*),* 4180. 10.1038/ncomms5180; 24939037

[86] Merz, F., Boehringer, D., Schaffitzel, C., Preissler, S., Hoffmann, A. et al. (2008). Molecular mechanism and structure of Trigger Factor bound to the translating ribosome. The EMBO Journal*,* 27 *(*11*),* 1622–1632. 10.1038/emboj.2008.89; 18497744 PMC2426727

[87] Ferbitz, L., Maier, T., Patzelt, H., Bukau, B., Deuerling, E. et al. (2004). Trigger factor in complex with the ribosome forms a molecular cradle for nascent proteins. Nature*,* 431 *(*7008*),* 590–596. 10.1038/nature02899; 15334087

[88] Lakshmipathy, S. K., Tomic, S., Kaiser, C. M., Chang, H. C., Genevaux, P. et al. (2007). Identification of nascent chain interaction sites on trigger factor. The Journal of Biological Chemistry*,* 282 *(*16*),* 12186–12193. 10.1074/jbc.M609871200; 17296610

[89] Jha, S., Komar, A. A. (2011). Birth, life and death of nascent polypeptide chains. Biotechnology Journal*,* 6 *(*6*),* 623–640. 10.1002/biot.201000327; 21538896 PMC3130931

[90] Patzelt, H., Rüdiger, S., Brehmer, D., Kramer, G., Vorderwülbeckem, S. (2001). Binding specificity of Escherichia coli trigger factor. Proceedings of National Academy of Sciences of United States of America*,* 98 *(*25*),* 14244–14249. 10.1073/pnas.261432298; 11724963 PMC64667

[91] Zhang, J., Bu, X., Wang, H., Zhu, Y., Geng, Y. et al. (2019). Cyclin D-CDK4 kinase destabilizes PD-L1 via cullin 3-SPOP to control cancer immune surveillance. Nature*,* 571 *(*7766*),* E10. 10.1038/s41586-019-1351-8 31270456

[92] Yamaguchi, I., Nakajima, K., Shono, K., Mizobuchi, Y., Fujihara, T. et al. (2019). Downregulation of PD-L1 via FKBP5 by celecoxib augments antitumor effects of PD-1 blockade in a malignant glioma model. Neuro-oncology Advances*,* 2 *(*1*),* vdz058. 10.1093/noajnl/vdz058; 32642723 PMC7212915

